# Reliability of optic disc edema area in estimating the severity of papilledema in patients with POEMS syndrome

**DOI:** 10.1186/s13023-020-01392-x

**Published:** 2020-05-19

**Authors:** Ling-shan Liu, Xiao Zhang, Hao Zhao, Xue-min Gao, Dao-bin Zhou, Rong-ping Dai, Jian Li

**Affiliations:** 1 Eight-year Program of Clinical Medicine, Peking Union Medical College Hospital, Peking Union Medical College, Chinese Academy of Medical Sciences, Beijing, China; 2 Department of Ophthalmology and Key Laboratory of Ocular Fundus Diseases|, Peking Union Medical College Hospital , Chinese Academy of Medical Sciences, No.1 Shuaifuyuan Wangfujing Dongcheng District, Beijing, 100730 China; 3 Department of Hematology, Peking Union Medical College Hospital , Chinese Academy of Medical Sciences, No.1 Shuaifuyuan Wangfujing Dongcheng District, Beijing, 100730 China

**Keywords:** POEMS syndrome, Papilledema, Vascular endothelial growth factor, Lenalidomide

## Abstract

**Background:**

Polyneuropathy, organomegaly, endocrinopathy, monoclonal gammopathy and skin changes (POEMS) syndrome is a rare paraneoplastic syndrome involving multisystem. Optic disc edema (ODE) is the most common ocular manifestation in patients with POEMS syndrome and serves as an independent prognostic factor. However, parameters previously used to estimate its severity were inconvenient and costly. This study was designed to bring forward a novel and practical parameter, optic disc edema area, to evaluate ODE in patients with this disease and applied it to assess effectiveness of lenalidomide combined with dexamethasone in respect of ODE.

**Results:**

Forty-one treatment-naive patients with POEMS syndrome were enrolled in this single-center prospective study and treated with lenalidomide combined with dexamethasone. They received ocular examination to determine optic disc edema (ODE) area and other optic manifestations. Meanwhile, serum VEGF was measured before and after treatment. Among 41 enrolled patients, 38 received complete ocular examinations, and 25 of which had ODE at initial visit. Binocular mean ODE area of patients with ODE was significantly related to ODE grade (r = 0.620, *p* = 0.003) and peripapillary retinal thickness (r = 0.760, *p* < 0.001) before treatment. Serum VEGF was significantly higher in patients with ODE than their counterparts (*p* = 0.025) and positively correlated with binocular mean ODE area (r = 0.460, *p* = 0.036). After treatment, ODE area, along with serum VEGF, decreased markedly (*p* < 0.001).

**Conclusion:**

ODE area was a reliable index to evaluate ODE severity and could precisely reflect ODE improvement through systemic treatment. Additionally, it was related to serum VEGF, a key factor in disease pathogenesis, suggesting its potential as an indicator of the overall severity of this disease.

**Trial registration:**

Clinicaltrials, NCT01816620. Registered March 222,013.

## Background

POEMS syndrome is a rare, chronic, multisystemic disease, characterized by acronym of a series of distinct features: polyneuropathy (P), organomegaly (O), endocrinopathy (E), monoclonal or M-protein band (M) and skin changes (S). Furthermore, some clinical signs beyond the extent of the definition of POEMS syndrome also contribute to the diagnosis of POEMS syndrome, of which papilledema is an important component. Among all reported ocular symptoms in POEMS syndrome patients, papilledema is the most common one and serves as a negative prognostic factor [1, 2].

The pathogenesis of POEMS syndrome and its presentation of ODE is still under study. However, vascular endothelial growth factor (VEGF) is considered to be a pivotal factor in the progressing of many clinical features [1], including papilledema. The connection between papilledema and serum VEGF has already been reported, both in our previous study and in studies of other ophthalmologists or hematologists, based on peripapillary retinal thickness (pRT) and subfoveal choroidal thickness [3–6]. But the measurements of these two parameters were inconvenient and costly, thus impractical for routine ODE assessment.

Therefore, we conducted this prospective study in our center to bring forward a novel convenient evaluation index, optic disc edema (ODE) area, and determine its reliability in reflecting ODE severity and treatment efficacies in patients with POEMS syndrome in terms of ocular manifestations, and besides, to further confirm the relationship between papilledema and serum VEGF level.

## Methods

### Patients

Forty-one newly-diagnosed patients with POEMS syndrome who met the diagnostic criteria defined by Dispenzeri were enrolled from April 2014 to November 2014 [7]. The diagnosis of POEMS syndrome, according to Dispenzeri, was established when two mandatory major criteria (polyneuropathy, monoclonal plasma cell-poliferative disorder), one of the other major criteria (Castleman disease, sclerotic bone lesions, VEGF elevation), and one of six minor criteria (organomegaly, extravascular volume overload, endocrinopathy, skin changes, papilledema, thrombocytosis/polycythemia) simultaneously occurred to a patient [1, 7]. Patients previously diagnosed with malignant tumor or treated with immunomodulatory drugs were excluded from the cohort. And patients with history in either eye of optic nerve diseases, glaucoma, diabetic retinopathy, retinal or choroidal vascular diseases, uveitis, high myopia no less than 6.00DS and who had undergone intraocular surgery were excluded either.

### Treatment

All enrolled patients were treated with 12 cycles of lenalidomide combined with dexamethasone (LDex treatment): lenalidomide (Revlimid; Celgene Corporation, Summit, NJ, USA) at a dose of 10 mg qd from d1–2121 and dexamethasone at a dose of 40 mg qw at d1,8,15,22 throughout the 28-day cycle. Besides, to prevent thrombosis, aspirin was prescribed at a dose of 100 mg per day.

### Ocular examination

All enrolled patients were supposed to receive complete ocular examination before treatment and every 3 months after the completion of the first treatment cycle until the completion of the total 12 cycles, complete remission of papilledema or death of patients. Best-correct visual acuity (BCVA) measured by the International Standard Visual Acuity Chart, intraocular pressure (IOP), pupillary reflect, ocular motility, slit lamp examination and fundus examination were performed. In addition, fundus photography (TOPCON TRC.NW6S, Non-mydriatic retinal camera), slit lamp examination with Volk 90D lens and spectral domain optic coherence tomography (SD-OCT, Spectralis OCT; Heidelberg Engineering, Heidelberg, Germany) were also performed at each visit. Among them, we placed emphasis on slit lamp examination with Volk 90D and fundus photography to determine the severity of papilledema.

#### ODE severity evaluation

Slit lamp examination with Volk 90D lens and fundus photography were performed at each visit to grade the extent of ODE and obtain the area of optic disc edema (ODE area).

**Fig. 1:**
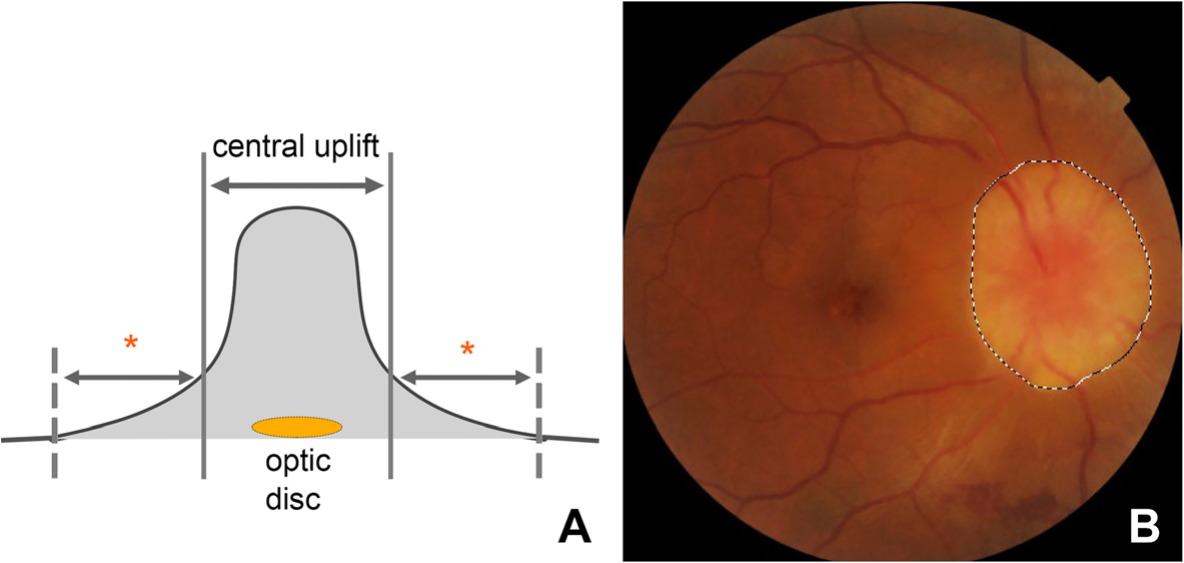
Determination of ODE area through fundus photography assisted by a stereoscopic vision. **a** illustration of the stereoscopic vision of edematous area around optic disc obtained by slit lamp examination with Volk 90D lens. **b** borderline of the ODE area on a fundus photograph of a patient’s right eye with grade 5 papilledema (all major vessels were obscure on the optic disc). The edematous area often manifested as a tall and steep central uplift and a surrounding gentle downslope (marked with asterisk) in stereoscopic vision as shown in A, with a clear boundary between severe and mild edematous area but a blurred boundary between mildly edematous area and completely normal retina. The borderline of ODE area we selected on fundus photograph shown in B was the area of severe edema, corresponding to the margin of central uplift in A, and was often located at the region where color changed from edematous grayish-white to yellow-gray most dramatically. In B, we could also see an obscure yellow-grey area peripheral to the ODE area we outlined. It is the mildly edematous area corresponding to the gentle downslope in A that was not counted in this measurement

(1) ODE grade evaluation. ODE grade was determined by ophthalmologists according to Modified Frisén Scale, in which the severity of ODE was stratified into 5 categories: Grade 1 (minimal degree), the appearance of a C-shaped halo of opacification of the RNFL at the region of the optic disc border; Grade 2, the presence of a circumferential halo with decreased translucency, whereas all major retinal vessels were visible throughout the optic disc and its border; Grade 3, segmentally loss of discontinuity in at least one major vessel when coursing over the optic disc margin; Grade 4, a broader discontinuity due to opacification in at least one but not all major vessels on the optic disc; Grade 5, obscuration of all major vessels on the optic disc [8]. Declination of the grade by one or more levels in either eye after treatment was considered as remission of ODE, while decreasing to zero in both eyes was considered as complete remission.

(2) ODE area measurement. Since ODE in patients with POEMS syndrome is caused by edema of tissues around optic disc instead of optic disc itself, ODE area in our study was defined as the area of obvious edematous zone around optic disc. The determination of its border was assisted by the stereoscopic vision from slit lamp examination with Volk 90D lens. In the stereoscopic vision, some patients’ edematous zone manifested as a central tall and steep uplift surrounded by gentle downslope gradually declining to normal retina (Fig. 1a). The border of ODE area was defined as the contours of the central steep uplift. Corresponding to the contours in stereoscopic vison, edematous area was outlined on the fundus photograph, and this margin was usually located at the region where color changed from edematous grayish-white to yellow-grey most dramatically, as shown in Fig. 1b. The edematous borderline of each patient was determined by the same experienced ophthalmologist and checked by a second one and blinded to serum VEGF level and peripapillary retinal thickness (pRT). In cases of disagreement, a superior ophthalmologist was requested. The area of this obviously edematous zone (ODE area) was measured automatically by the self-contained area calculation program in the retinal camera (TOPCON TRC.NW6S).

### Serum VEGF level measurement

Human Quantikine ELISA Kit (R&D Systems, Minneapolis, MN, USA) was used to measure serum VEGF levels at baseline before the first treatment cycle and during follow-up, monthly after each cycle during first 3 months, every 3 months afterwards until 6 months after the whole treatment course. Besides, ocular examination was performed within 3 days after VEGF measurement, though not at every follow-up in the first 3 months. Normal range of VEGF was considered as below 600 pg/mL [9].

### Statistical analysis

SPSS statistics 17.0 (SPSS Inc., Chicago,IL, USA) was used to perform statistical analysis. Paired t test and intraclass correlation coefficient (ICC) were utilized to judge the consistency of ODE area in right and left eyes. The comparison of continuous variables including serum VEGF and ODE area between different groups was based on independent-sample Student t test. The correlation between ODE area and ODE grade was determined by Spearman correlation coefficient, while correlations between ODE area and serum VEGF as well as between ODE area and peripapillary retinal thickness (pRT, data extracted from our former study [6]) were performed using Pearson correlation coefficient. *P* value less than 0.05 was considered as statistically significant.

## Results

### General information at baseline

At initial visit, all 41 newly-diagnosed patients had polyneuropathy, endocrinopathy, M-protein band and skin changes. Organomegaly manifested mostly as lymphadenopathy (38/41, 92.7%). Besides, splenomegaly (27/41, 65.9%) and hepatomegaly (24/41, 58.5%) were not uncommon. Endocrinopathy included 71.8% (28/41) of increased ACTH, 41.5% (17/41) of hypothyroidism, 14.6% (6/41) of diabetes mellitus, and 75% (21/28) of male gynecomastia. Thirty-six patients completed 12 cycles of LDex treatment, while 3 patients died due to disease progression and 2 quitted the clinical trial midway. In this study, three patients who did not meet the basic requirement of ocular examination were excluded: one patient with incomplete initial ocular examination data, one with missed pre-treatment ODE area data, and another one with delayed first posttreatment measurement of optic disc at 12 months later. At diagnosis, average age of the enrolled 38 patients with complete data was 50.0 ± 11.5 years (range 21–70). Among them, 26 were male and 12 were female. Mean serum VEGF level was 5655 ± 3290 pg/mL (range 534–14,328).

### Ocular manifestations and ODE grading before and after treatment

At the first ocular examination before treatment, 25 (65.8%) patients were diagnosed with ODE, of which 24 (96%) were bilateral and only 1 (4%) was unilateral. ODE stratification was determined in these 25 patients: 1 eye (2%) was grade 0, 12 eyes (24%) were grade 1, 10 eyes (20%) were grade 2, 11 eyes (22%) were grade 3, 14 eyes (28%) were grade 4 and 2 eyes (4%) were grade 5. Among the 25 patients with ODE, except 8 (32%) patients were unsymptomatic, the other 17 patients (68%) had various symptoms including blurred vision (7/17, 41.2%), decreased vision (4/17, 23.5%) and palpebral edema (3/17, 17.6%). Red eye, photophobia and photopsia also appeared but relatively rare. In contrast, all the other 13 patients (34.2%) without ODE did not show any ocular symptoms at baseline or during follow-up.

**Fig. 2:**
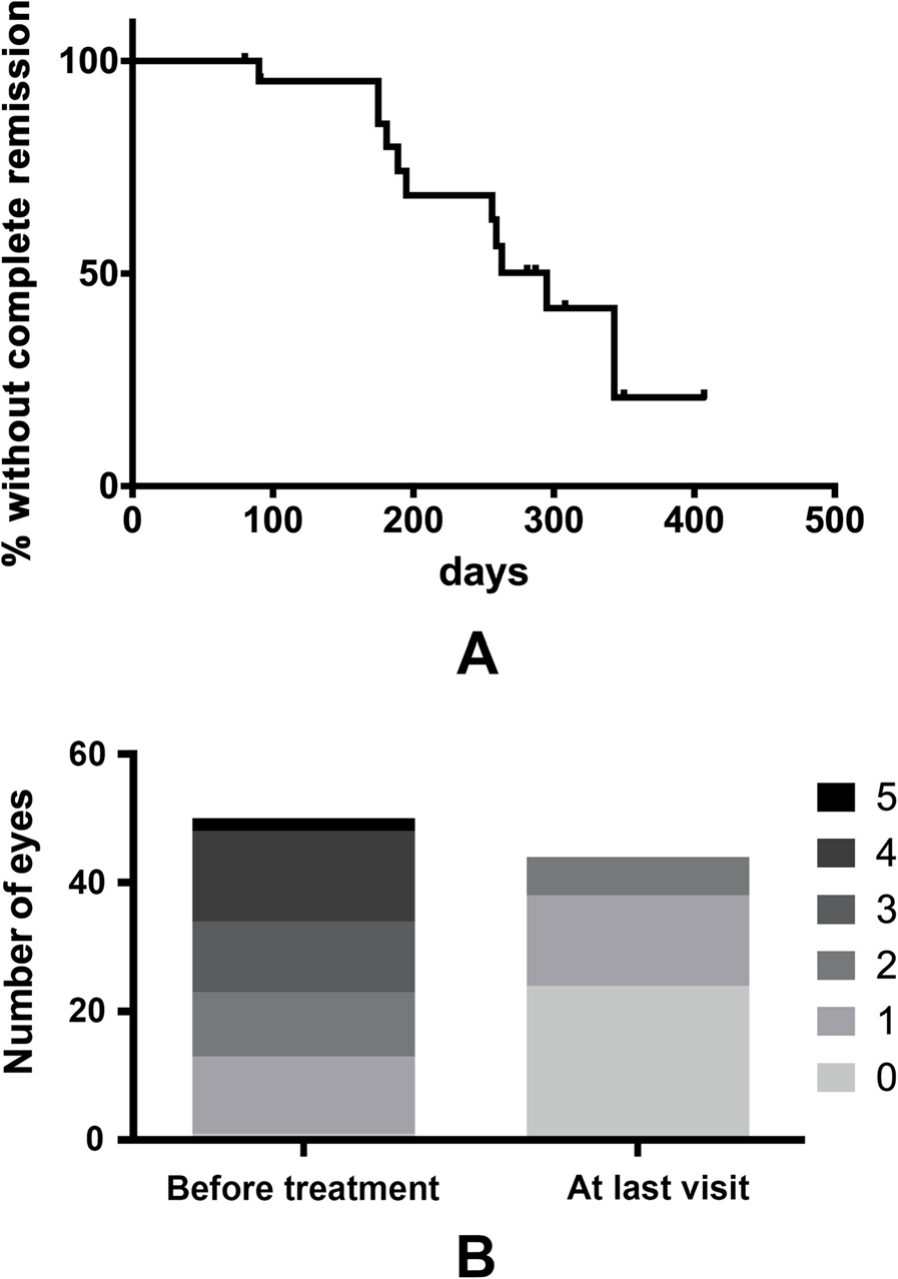
ODE grade declination through treatment. **a** Kaplan-Meier plot showing the complete remission of ODE defined by ODE grade decreasing to 0. Through the follow-up of a mean duration of 8.6 ± 3.3 months (range 3–13), the complete remission rate at last visit was 54.5%. **b** distribution of ODE grade before and after treatment. Before treatment, ODE grade was 0 in 1 eye (2%), 1 in 12 eyes (24%), 2 in 10 eyes (44%), 3 in 11 eyes (22%), 4 in 14 eyes (28%) and 5 in 2 eyes (32%). In comparison, after treatment, ODE grade was 0 in 24 eyes (54.2%), 1 in 14 eyes (33.3%) and 2 in 6 eyes (12.5%)

**Fig. 3:**
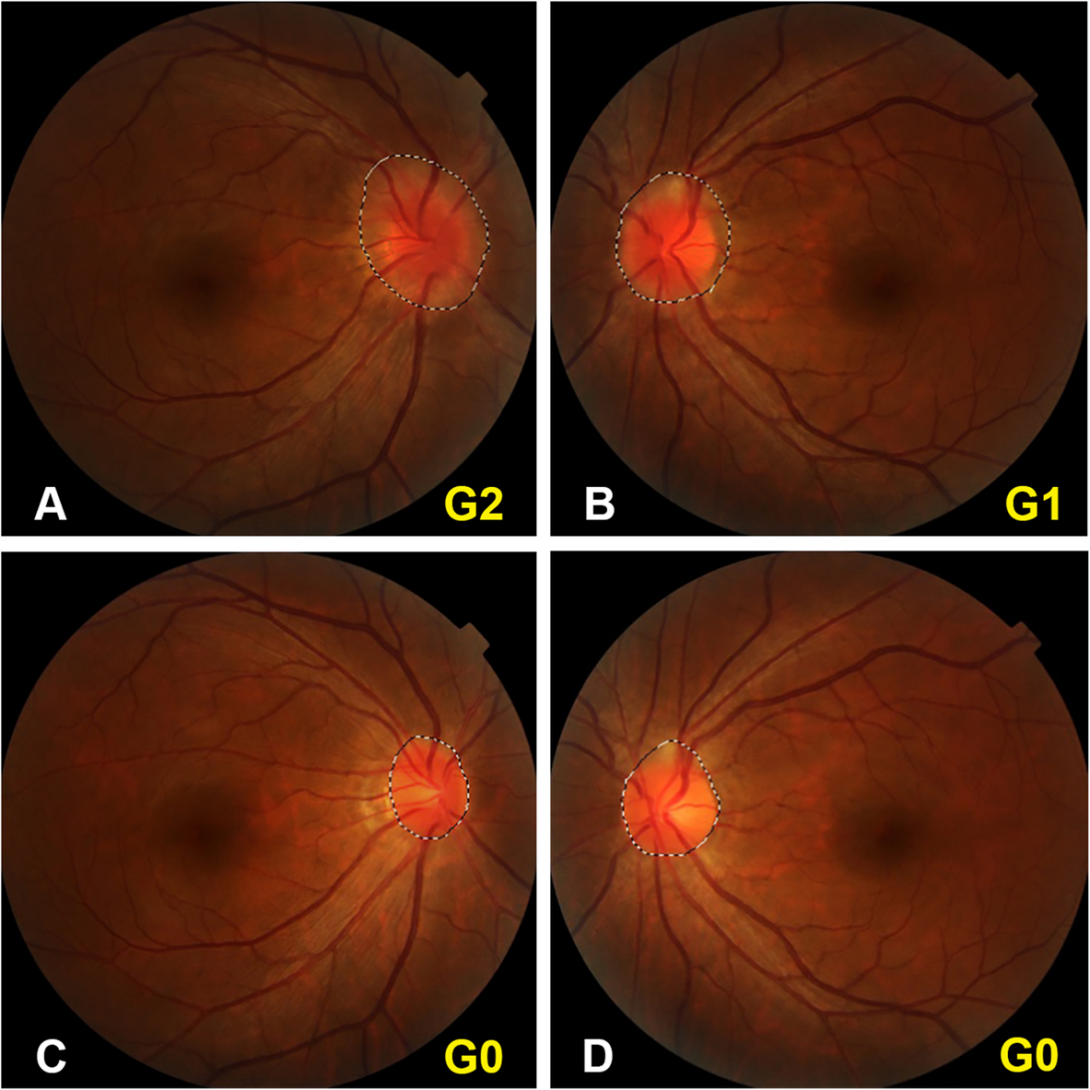
Fundus photography showing ODE improvement of an individual POEMS syndrome patient through treatment. Male, 42y, ODE was significant before treatment, with grade 2 in right eye (**a**) and grade 1 in left eye (**b**). A circumferential translucent grey area around optic disc was seen in right eye, while a c-shaped semi-opaque grey area wrapping the optic disc from temporal and upper sides was seen in left eye. All major vessels in both eyes were clear and distinct. Serum VEGF was 5922 pg/mL before treatment. After 10-month treatment, ODE was completely recovered in both eyes (**c** right eye, **d** left eye). Serum VEGF was 1547 pg/mL. ODE area of both eyes decreased markedly after treatment

**Fig. 4:**
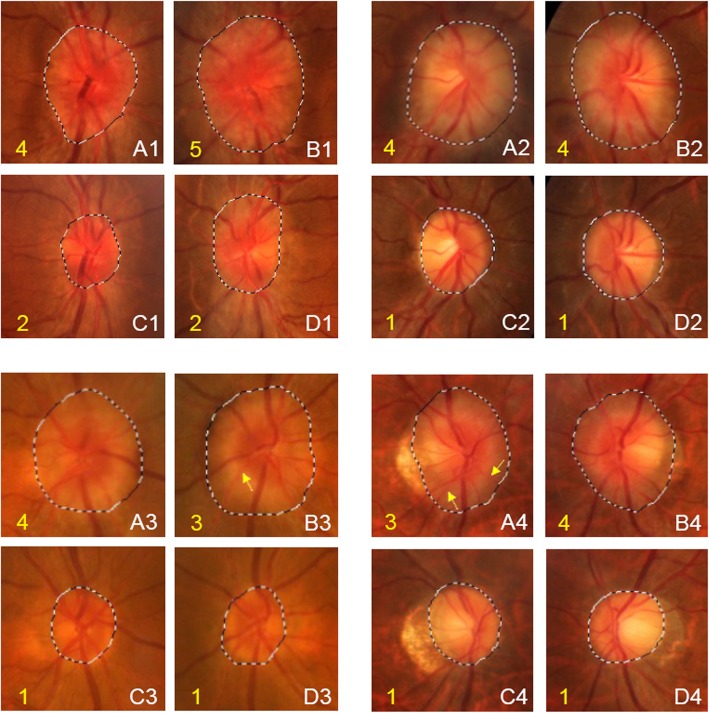
Fundus photography indicating ODE area and ODE grade change through treatment. Four patients’ fundus photographs with amplification of edematous optic disc was shown in the Figure (**a** right eye before treatment, **b** left eye before treatment, C. right eye after treatment, D. left eye after treatment), ODE grades were marked with yellow number. Compared to optic disc regions before treatment, remarkable declination of ODE area after treatment were seen in both eyes of these 4 patients. Before treatment, ODE grades of these four patients’ eyes were ranged 3–5. ODE in A2 was grade 5, with obscuration of all major vessels on the optic disc. ODE in B1, A2, B2, A3 and B4 were grade 4, with relatively wide discontinuity of the major vessels at different areas surrounding optic disc: A1, B2 at nasal side; A2 at three quadrants except the inferior temporal quadrant; A3, B4 at superior nasal quadrant. ODE in B3 and A4 were grade 3, with only segmental discontinuity of vessels at margin of optic disc marked with yellow arrows. After treatment, except both eyes of the first patient still had circumferential opaque area and were rated grade 2 ODE, all the other patients’ eyes’ ODE grades declined to 1. C-shaped halo of opacification in each patient’s eye was at different sides of optic border: C2, nasal, superior and inferior side; D2, nasal and inferior side; C3, temporal side; D3, nasal, superior and inferior side; C4, nasal, superior and inferior side; D4, temporal and inferior side. The changes of ODE area and grade convincingly prove the effectiveness of lenalidomide in terms of ocular symptom

All enrolled patients received treatment with LDex after initial examination. Except for 3 patients who either died during treatment or showed very slight ODE and refused to receive follow-up ocular examination, all the other 22 patients had ocular examination follow-up, with a mean duration of 8.6 ± 3.3 months (range 3–13). Twenty-one patients received ocular examination 3 months after treatment. The remission rate was 83.3% (35/42) and the complete remission rate was 14.3% (6/42), with only 7 eyes remained stable ODE grade. At last visit, the remission rate reached 90.9% (40/44) and the complete remission rate reached 54.5% (24/44). ODE grade was 0 in 24 eyes (54.2%), 1 in 14 eyes (33.3%) and 2 in 6 eyes (12.5%) (Figs. 2, 3, and 4). Ocular manifestations disappeared in almost all patients at last visit except for one patient who had remnant blurred vision even with complete remission of ODE.

### ODE area measurement before and after treatment

ODE area was measured in 21 patients with ODE at initial examination. Except one patient died during treatment, all the other 20 patients had their ODE areas measured at follow-up ocular examinations.

The ODE area of right eye and left eye at initial examination was 2.0098 ± 0.8050 mm^2^ (range 0.8064–3.2659) and 1.9310 ± 0.7747 mm^2^ (range 0.8124–3.8495) respectively. There was no significant difference between ODE area of right and left eyes (*p* = 0.595), and there was a relatively good consistency between them, with an intraclass correlation coefficient (ICC) of 0.652 (*p* < 0.001). Binocular mean ODE area was 1.9704 ± 0.7158 mm^2^ (range 0.8094–3.5577). There was a significant correlation between ODE area and ODE grading before treatment (right eye, r = 0.632, *p* = 0.002; left eye, r = 0.638, *p* = 0.002; binoculus, r = 0.620, *p* = 0.003).

**Fig. 5:**
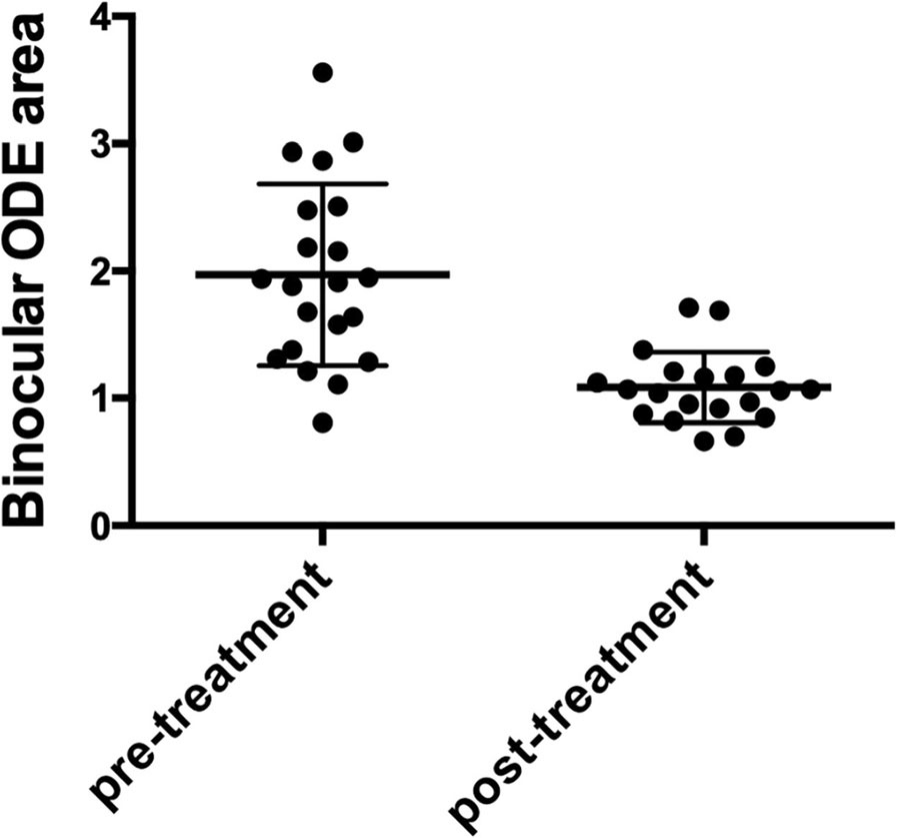
ODE area decreasing in POEMS syndrome patients with ODE through treatment. Binocular mean ODE area was 1.9704 ± 0.7158 mm^2^ (range 0.8094–3.5577) at initial visit before treatment. After treatment, it decreased significantly to 1.0839 ± 0.2768 mm^2^ (range 0.6646–1.7118) at last visit (*p* < 0.001)

At last visit, ODE area of right and left eye of the 20 alive patients was 1.0501 ± 0.2760 mm^2^ (range 0.6395–1.7315) and 1.1176 ± 0.3265 mm^2^ (range 0.6690–1.9767) respectively, without significant difference (*p* = 0.230). Binocular mean ODE area was 1.0839 ± 0.2768 mm^2^ (range 0.6646–1.7118), which was remarkably lower than baseline (*p* < 0.001). The declination in ODE area was noticeable through treatment (Fig. 4, and 5).

### Serum VEGF levels and ODE area

As stated above, mean serum VEGF level of all 38 POEMS patients with or without ODE at initial visit was 5655 ± 3290 pg/mL (range 534–14,328). And 3 months post-treatment, serum VEGF level of 37 alive patients declined to 1979 ± 2132 pg/mL (range 181–11,179), significantly lower than baseline VEGF (*p* < 0.001).

At initial visit, baseline serum VEGF level in patients with and without ODE were significantly different, with a mean level of 6564 ± 3257 pg/mL (range 1553–14,328) in patients with ODE and 3907 ± 740 pg/mL (range 534–8594) in patients without ODE, respectively (*p* = 0.016).

**Fig. 6:**
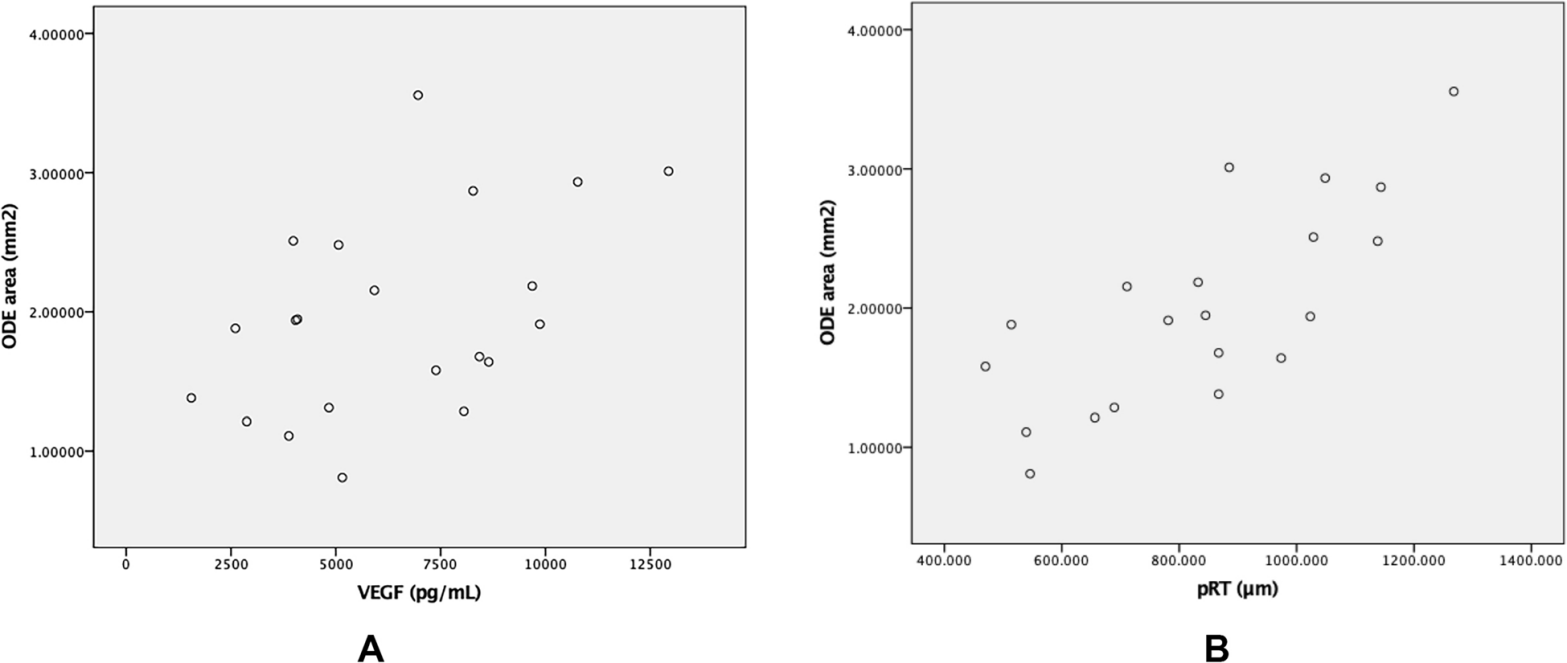
Correlation between ODE area and serum VEGF as well as peripapillary retinal thickness (pRT). **a** correlation between serum VEGF and ODE area. Binocular mean ODE area of POEMS syndrome patients with ODE was significantly correlated with serum VEGF before treatment (r = 0.460, *p* = 0.036). **b** correlation between ODE area and pRT. Binocular mean ODE area of POEMS syndrome patients with ODE was significantly correlated with pRT before treatment (r = 0.760, *p* < 0.001)

Besides, binocular mean ODE area was positively correlated with serum VEGF among patients with ODE at diagnosis (r = 0.460, *p* = 0.036, Fig. 6a). However, the correlation after treatment was not significant (r = 0.015, *p* = 0.937).

### ODE area and peripapillary retinal thickness (pRT)

Since pRT was also supposed to be capable of indicating ODE severity, we did correlation analysis between ODE area and pRT among 20 patients with both two parameters measured at baseline, and the result confirmed the consistency between these two indicators of ODE severity (r = 0.760, *p* < 0.001, Fig. 6b). The measurement of pRT in these patients was described in one of our former published articles [6].

## Discussion

POEMS syndrome is a rare paraneoplastic syndrome involving multisystem due to underlying plasma cell disorder. According to previous studies, ocular signs and symptoms are common in POEMS syndrome, and ODE occurs most frequently, serving as an adverse prognostic factor of overall survival [2]. Consistent with existing reports, ODE was also the most common ocular finding in our trial, with an incidence of 65.8%, close to the upper bound of previous data (30–70%) [1, 2, 6, 7, 10, 11]. Analogous to the previous study [11], 68% of patients with ODE showed variable associated symptoms at initial examination, including blurred vision, decreased vision, palpebral edema, red eye, photophobia and photopsia.

The pathogenesis of POEMS syndrome is complex and remains contested. However, VEGF, as a cytokine increasing vascular permeability reversibly and promoting angiogenesis, is considered as a potential contributor and correlated with disease activity, serving as an important prognostic factor [12–15]. It has also been suggested that elevated serum VEGF level might be a cause of ODE in POEMS syndrome [3, 4, 6, 10]. The relationship between ODE and serum VEGF in this disease has been proved in our recent studies based on binocular mean peripapillary retinal thickness (pRT) and retinal nerve fiber layer (RNFL) thickness [6]. However, pRT and RNFL thickness are significantly affected by several factors including myopia and optic disc size [16–18]. Besides, their measurements are costly and not easily accessible enough to make them routine ocular tests for POEMS patients. Thus, as an extension, we chose ODE area as an evaluation parameter in this current study. In our study, ODE area was significantly correlated to ODE grade before treatment. The correlation between ODE area and pRT extracted from our previous study was prominent too [6]. Thus, these two parameters were consistent and both efficacious in reflecting the severity of ODE. In addition, relationship between ODE area and serum VEGF before treatment was remarkable. This not only confirmed the relationship between VEGF and ODE in POEMS syndrome, but also indicate the possibility of ODE area to approximately represent patients’ serum VEGF level.

Besides ODE, edema also appeared elsewhere in our patient cohort, including lower extremities, abdomen and pleura. Thus, ODE was more like an ocular manifestation of systemic edema caused by circulating serum VEGF rather than a focal symptom caused by tissue-borne VEGF [10, 19]. It has been reported that the effect of VEGFA on increasing vascular permeability could only occur when it binds to VEGFR2, which is predominantly located at abluminal side of microvascular endothelium and is rarely seen at luminal side [20]. Studies have shown that choroidal vessels, in comparison to retinal vessels, may be more susceptible to serum VEGF level due to its larger caliber and flow volume. Circulating VEGF in choroidal vessels is more likely to leak out, binding to abluminal VEGFR2 to improve permeability of surrounding vessels [6, 20, 21]. Besides, there is an area with a defective blood-retinal barrier surrounding the optic disc, called the tissue of Elschnig border, where substances in choroidal vessels could leak out even more easily. Thus, ODE in POEMS syndrome is probably the result of choroidal edema caused by high serum VEGF level [4]. Thus, ODE in patients with POEMS syndrome, triggered by circulating high level of VEGF, could only be relieved completely through systemic treatment. Local anti-VEGF treatment with bevacizumab could release ODE to some extent. However, ODE often recurs after ceasing local treatment, necessitating a more thoroughly clearance of monoclonal plasma cells by systemic treatment. The outcome of systemic treatment with LDex was satisfactory in our study: ODE was relieved in 90.9% and completely resolved in 54.2% of patients at last visit, accompanied by a significantly reduction of ODE area and serum VEGF level. Although correlation between VEGF and ODE area before treatment was significant, the correlation coefficient was 0.460, indicating other factors might contribute to ODE generation. Elevated intracranial pressure (ICP), vasculitis and nerve infiltration were three other reported causes [10, 11], among which elevated ICP received most attention. However, relevant results were controversial: some studies affirmed the correlation between elevated ICP and ODE [2, 22], while some others repudiated it [3, 23]. Unfortunately, ICP were recorded in only seldom patients in our cohort thus it was unpractical to include it in our study. Another limitation was incomplete measurement of choroidal thickness due to obscurity of choroid borders in many patients with ODE, making it impossible to verify the presumed ODE pathogenic mechanisms in POEMS syndrome stated above. In addition, optic disc area changes in different ethnic groups, axial length and myopic refractive error [24, 25], yet this bias could not be avoided since it was hard to determine original disc area in patients with ODE. Beyond that, considering the intra-subject inter-eye dependencies, treating both eyes of a patient as an integral could lead to inaccurate results. However, ODE in POEMS syndrome is part of a systemic pathological conditions and usually occurs evenly in both eyes. Also, none of the patients in this trial have amblyopia and other diseases that could cause significant differences in binocular optic disc area. ICC between ODE area of right and left eyes shows a quite good consistency, further ensuring the credibility of this analysis. Despite these limitations, this is still a valuable work. On the one hand, we brought forward ODE area as an evaluation index to estimate the severity of ODE in a relatively large cohort of POEMS patients, and outcomes proved its reliability. Besides, ODE area was significantly correlated to serum VEGF. Since fundus photography is more accessible than SD-OCT and ODE area measurement is more convenient and less expensive than pRT and RNFL thickness measurement, ODE area could act as a feasible parameter of evaluating the change of ODE as well as VEGF level in patients with POEMS syndrome. On the other hand, we confirmed the effectiveness of LDex treatment in terms of ocular involvement.

## Conclusison

ODE area was an intuitive and effective index in ODE evaluation which is expected to put into clinical practice in patients with POEMS syndrome. Besides, we confirmed that ODE, related to serum VEGF level, could be resolved successfully through systemic treatment with lenalidomide combined with dexmethasone.

## Data Availability

The dataset supporting the conclusions of this article is included within the article.

## Abbreviations

POEMS syndrome: Polyneuropathy, organomegaly, endocrinopathy, monoclonal gammopathy and skin changes syndrome
ODE: Optic disc edema
ODE area: Optic disc edema area/edematous optic disc area
VEGF: Vascular endothelial growth factor
pRT: Peripapillary retinal thickness
RNFL: Retinal nerve fiber layer
BCVA: Best-correct visual acuity
IOP: Intraocular pressure
SD-OCT: Spectral domain optic coherence tomography
ICP: Intracranial pressure
